# Supplementary material for: Protocol for an open labelled observational study in children & adolescents with severe asthma with an eosinophilic phenotype treated with mepolizumab (CASAM)
Source: PLoS One. 2025 Dec 19;20(12):e0335399. doi: 10.1371/journal.pone.0335399 (PMC12716689; doi:10.1371/journal.pone.0335399)
Supplement: Attachment — Submitted filename: Response to Reviewers[37][5].docx [file pone.0335399.s003.docx]

Date: 25/09/2025

Dear Editor and Reviewers,

We thank the editors and reviewers for their thoughtful comments, which have helped us improve the clarity and quality of our manuscript. We have addressed each point in detail below.

*4. Have the authors described where all data underlying the findings will be made available when the study is complete?

The* [*PLOS Data policy*](https://eur03.safelinks.protection.outlook.com/?url=https%3A%2F%2Ftrack.editorialmanager.com%2FCL0%2Fhttps%3A%252F%252Fjournals.plos.org%252Fplosone%252Fs%252Fmaterials-and-software-sharing%2F1%2F010f01990a612e3a-ad461904-83eb-491f-b743-145ea400240c-000000%2FMaTJpVPmIgYEAUGPS5gdoGxVSM4eF6g-AyLc5yUaYos%3D226&data=05%7C02%7Clatika.gupta%40kcl.ac.uk%7C45d56bcb63ec4a574fc308ddeac26d82%7C8370cf1416f34c16b83c724071654356%7C0%7C0%7C638924842977116856%7CUnknown%7CTWFpbGZsb3d8eyJFbXB0eU1hcGkiOnRydWUsIlYiOiIwLjAuMDAwMCIsIlAiOiJXaW4zMiIsIkFOIjoiTWFpbCIsIldUIjoyfQ%3D%3D%7C0%7C%7C%7C&sdata=sIraOqqnMhluayevQ36dEjZzHBB6CUpbQLOReLBIpxg%3D&reserved=0) *requires authors to make all data underlying the findings described in their manuscript fully available without restriction, with rare exception, at the time of publication. The data should be provided as part of the manuscript or its supporting information, or deposited to a public repository. For example, in addition to summary statistics, the data points behind means, medians and variance measures should be available. If there are restrictions on publicly sharing data—e.g. participant privacy or use of data from a third party—those must be specified.*

*Reviewer #1: No*

*Reviewer #2: Yes*

We thank the reviewers for their comments. To address this, we have expanded our Data Availability Statement in the protocol.

*List of changes made in protocol:*

1. *Section 4.7 Data Management:*

- *Data availability: All data of the findings of this study will be made available at the time of publication, either within the manuscript and its supporting information files. Where restrictions apply (e.g., participant confidentiality), these will be clearly specified.*

The data will be provided as supporting information at the time of publication.

*6. Review Comments to the Author

Please use the space provided to explain your answers to the questions above and, if applicable, provide comments about issues authors must address before this protocol can be accepted for publication. You may also include additional comments for the author, including concerns about research or publication ethics.

You may also provide optional suggestions and comments to authors that they might find helpful in planning their study.


(Please upload your review as an attachment if it exceeds 20,000 characters)*

*Reviewer #1: This protocol presents a multictre observational study that will evaluates the management of severe eosinophilic asthma in children and adolescents with mepolizumab. The introduction states the burden of paediatric severe asthma and the limited trial data on mepolizumab for children and adolescents. The primary objective is to assess the reduction in clinically significant exacerbations pre- and post-treatment. Methodologically, the study is feasible and technically sound.
While data management and long-term storage procedures are detailed, a data-sharing plan (eg repository, conditions of access, timeline) has not been specified.
In the exclusion criteria I believe that the following should be included:
1)Known hypersensitivity to mepolizumab or excipients.
2)Active clinically significant infection, eg untreated helminth/parasitic infection
3)Other primary pulmonary diseases that may influence outcomes according the investigator (eg cystic fibrosis, primary ciliary dyskinesia, interstitial lung disease)
4)Eosinophilic conditions with different indications
5)Concomitant biologic therapy for asthma or recent exposure without washout
Finally, the severity of the asthma exacerbations (mild, moderate, severe) should be a secondary objective*

*Reviewer #2: I have reviewed the "Protocol for an open labelled observational study in children & adolescents with severe asthma with an eosinophilic phenotype treated with mepolizumab (CASAM)" which is regarding a study initiated in March 2022 and likely to complete in March 2026. The study primarily aims to establish the efficacy of mepolizumab in eosinophilic severe asthma patients in the age group 6-17 years. It is an open label, real world observational study.
It is a very well written protocol especially designed to meet ethical issues that may arise in this population. All the aspects of methodology have been described in sufficient detail and utilize contemporary tools. Considering its scientific rigour, study will generate valuable data.*

We thank the reviewers for their comments.

We would like to clarify that, regarding the exclusion criteria mentioned above, the therapy (Mepolizumab) was initiated in accordance with national guidelines, specifically the NICE/BTS guidelines from the United Kingdom and the GINA guidelines in Spain. Before initiating a biologic therapy, the guidelines recommend ruling out other chronic respiratory, eosinophilia and helminthic causes. The guidelines and Mepolizumab product license recommend hypersensitivity review before initiation.

The NICE/BTS and GINA/ERS guidelines in children do not recommend the use of concomitant biologic therapies.

Being a real-world study, washout periods were not included in the exclusion criteria, but were as per national guidelines NICE/BTS guidelines from the United Kingdom and the GINA guidelines in Spain

Regarding the severity of exacerbations (mild, moderate, severe), this has been added as a secondary objective. The data is being collected and entered as per these categorisations.

List of changes made in protocol:

1. Section 3.2 Secondary Objectives:

- *Compare the rate of mild and moderate clinically significant exacerbations in the pre-exposure and the 12-month and 24-month post-exposure periods with mepolizumab.*
- *Compare the rate of severe asthma exacerbations requiring an ED visit in the pre-exposure and the 12-month and 24-month post-exposure periods with mepolizumab.*
- *Compare the rate of severe asthma exacerbations requiring hospitalization in the pre-exposure and the 12-month and 24-month post-exposure periods with mepolizumab.*

2. Section 4.5 Outcome evaluation:

- *Rate of clinically significant exacerbations (mild, moderate and severe) (24-month post-exposure): Evaluating the longer-term impact of mepolizumab treatment.*
- *ED Visits (12-month and 24-month post-exposure): Measuring the frequency of severe asthma exacerbations requiring emergency department visits.*
- *Hospitalizations (12-month and 24-month post-exposure): Assessing the rate of severe asthma exacerbations leading to hospital admissions.*

We trust that these revisions address the reviewers’ concerns, and we are grateful for the opportunity to improve our work. We hope the manuscript is now suitable for publication in the PLOS journal.

Many thanks

Atul Gupta
